# Women in chemistry: Q&A with Dr Aude Sadet

**DOI:** 10.1038/s42004-024-01323-y

**Published:** 2024-10-28

**Keywords:** Diagnostics

## Abstract

Dr Aude Sadet is a junior scientist who conducts research in the Biophysics and Biomedical Applications Laboratory at the Extreme Light Infrastructure of the National Research Institute for Physics in Magurele, Bucharest, Romania.

Aude graduated with a B.Sc. and a Master’s degree in Biomedical Sciences from Université Paris Cité. She has her Ph.D. from Ecole Normale Supérieure in Paris and Sorbonne University, where she introduced new methods based on hyperpolarized nuclear magnetic resonance (NMR) biomarkers to observe the function of enzyme cascades in real time. She moved to Romania in 2017 for a postdoc at the University of Bucharest. She then joined the biophysics team of the high-power laser department in the nuclear physics institute in Bucharest. In Romania, she developed new highly-sensitive methods for the detection of biomolecular interactions via 2D hyperpolarized NMR spectroscopy and protein structure using persistent NMR-observable states.

**Figure:**
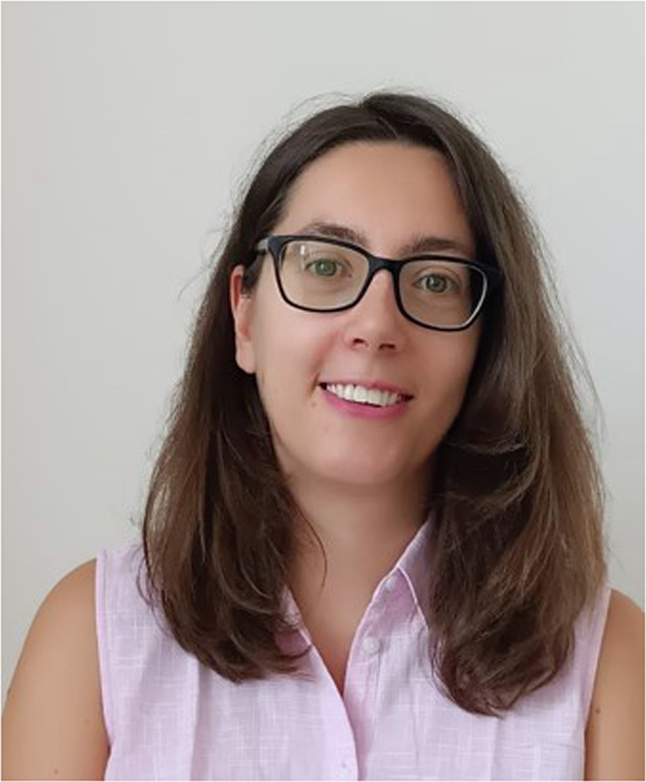
Aude Sadet

Aude Sadet was nominated for the Varian Prize at the Experimental Nuclear Resonance Conference, California, 2019. Her present research is interdisciplinary: developing new NMR methods to observe the effects of high dose-rate radiation in cells. The goal is to improve functional diagnostic of treatment effects in oncology.

Why did you choose to be a scientist?

Science has attracted and continues to captivate me, especially in light of medical applications. When I was young, I watched the series “Once upon a time… Life”. I really loved these series. And I think my passion for medical sciences started at that time. I have always been fascinated by how the human body works and by the process of healing. I see the living body as its own world. From a macroscopic point of view, each organ is different and has its own functions. From a microscopic point of view, organs are composed of cells that have very similar molecular mechanisms: the same constituents can be found, albeit with different concentrations, in every type of tissue; similar enzymes are at work, yet with different activities.

On a close look, a body functions like a beehive. Every part has its function regulated by some molecular machines. However, from one person to another, we do not react in the same way to various types of disease, nor do we respond in the same way to treatment. I had to deal with these aspects at a very personal level. My mother died of cancer when I was still a child. I accompanied her to chemotherapy and radiotherapy sessions and I remember that the diagnosis of her cancer was very late. I think this influenced my career directions.

What scientific development are you currently most excited about?

I am interested in many aspects of biochemistry applied to health sciences, especially in molecular research. For instance, I look forward to see the full reach of applications of mRNA vaccines in oncology. In my research, the applications of molecular mechanisms I aim to understand range from the development of new diagnostic methods by NMR to new types of treatment.

What direction do you think your research field should go in?

In my opinion, to accelerate progress in biomedical research, we need a timely and individualized diagnostic method. This necessity has been highlighted by the recent pandemic, when fast validation of new treatment was the main issue. Functional diagnostics become increasingly important as effective treatments in oncology turn out to rely on molecular mechanisms that are both complex and individualized, and thus molecular diagnostics have to be able to evaluate treatment according to every patient’s metabolism.

Do you have any advice you would like to share with women starting out in chemical research?

Throughout our career we have to make choices, particularly between our professional and personal lives. I would like to tell my women colleagues to follow their heart; whatever our decisions are, someone might criticize or question them. Therefore, I would encourage women in science to take advantage of the opportunities offered to them and, above all, stay in tune with themselves!

What steps do you feel are needed to retain more women in chemistry, for example from PhD level to Professorship?

The quality of the selection process in academia depends on the quality of decision-makers. True creativity and diversity is not directly reflected by bibliometrics or other criteria introduced as a fix. A transparent and thorough selection of highly-qualified individuals for positions of responsibility can improve the selection process for women. High standards to elect decision-makers would have to be backed by a public agenda, submitted to academics for debate. These decision-makers would then be accountable for the creativity of selected researchers, for choosing individuals with a true history of engaging in new challenges, and for how women fare in the selection process.

What impact has your gender had on your career as a scientist?

I am a mother and my son needs my support, especially during his first years. This has not impacted my research goals, since my expertise and creativity had been shaped during my studies and early work experience. However, I became aware that certain procedures and metrics are divergent with the normal life of a woman scientist. There are institutional procedures and practices that are contrary to both innovation and family life, and it is surprising that such practices persist in the modern inter-connected society.

Could you describe a memorable moment in your career where being a woman made a significant difference, possibly even steered your path in a certain direction?

I wish there had been moments when being a woman could have had a positive impact on my career, yet so far I have only seen hurdles in the career path related to this. There is a very revealing example of a national research project I applied for, where one of the anonymous reviewers chose, in sharp contrast with the other reviewers, to score according to arguments that were strongly biased against women. I keep a blog on such circumstances and their outcome at goeastyoungwomenscientists.wordpress.com. Even when there are EU norms and laws in place protecting the status of women researchers, in practice outdated procedures are used for the disavowal of women researchers.

Has geographical location or specific institute membership played a role in your experience as a woman in chemistry?

Eastern Europe can become a very good place for science, as high education standards were the tradition and the inherent local resilience has built motivated researchers who are often also very creative in tackling scientific challenges. However, research during the communist regime remained isolated from many scientific directions that developed in the west. The gap could not be quickly recovered because of inertia. A thick wall of bureaucracy is used to keep the status quo developed within circles of interests. Top-down and micromanagement practices create a ceiling for young researchers who try to develop new ideas. Efforts to introduce modern research techniques are often met with hurdles for financing and fluctuating institutional support. The directions of research at the interface between chemistry, physics, and biology still need to cross these barriers that are both cultural and geographic in nature.

How can research institutions and funders better support women scientists?

Funding institutions need to treat women scientists ethically in order for us to be able to continue research. Both EU and national legislation are in place for protecting the equilibrium between personal lives and researcher’s careers. This equilibrium is one of the key factors for researchers’ creativity. Unfortunately, in many cases, regulations do not follow the EU legislation. Some of the decision-makers choose to grant rights provided by legislation to women only *in extremis*, or not at all. Even in a state where declining natality rates and low life expectancy are known problems, there are public institutions that discourage healthy work-life balance. A very simple action for the people in charge would be to harmonize the rules and the spirit of their organization with existing legislation, including the legal and ethical standards regarding women’s rights.

*This interview was conducted by the editors of Communications Chemistry*.

